# Author Correction: Photonic crystals with rainbow colors by centrifugation-assisted assembly of colloidal lignin nanoparticles

**DOI:** 10.1038/s41467-024-46227-6

**Published:** 2024-02-28

**Authors:** Jinrong Liu, Mathias Nero, Kjell Jansson, Tom Willhammar, Mika H. Sipponen

**Affiliations:** 1 Department of Materials and Environmental Chemistry, Stockholm University, SE-106 91, Stockholm, Sweden; 2 Department of Materials and Environmental Chemistry, Wallenberg Wood Science Center, Stockholm University, SE-10691, Stockholm, Sweden

**Keywords:** Nanoparticles, Photonic crystals, Colloids

Correction to: *Nature Communications* 10.1038/s41467-023-38819-5, published online 29 May 2023

The original version of this Article contained an error in Equation 3. The equation contained an additional root symbol and incorrectly read:

$${n}_{{{{\rm{eff}}}}}=\sqrt{\varphi {n}_{{{{\rm{lig}}}}}+\left(1-\varphi \right){n}_{{{{\rm{air}}}}}}$$

on page 7 of the manuscript file.

The correct form of equation 3 is:

$${n}_{{{{\rm{eff}}}}}=\varphi {n}_{{{{\rm{lig}}}}}+\left(1-\varphi \right){n}_{{{{\rm{air}}}}}$$

This has been corrected in the PDF and HTML versions of the Article.

