# Supplementary material for: Photonic crystals with rainbow colors by centrifugation-assisted assembly of colloidal lignin nanoparticles
Source: Nat Commun. 2023 May 29;14:3099. doi: 10.1038/s41467-023-38819-5 (PMC10227086; doi:10.1038/s41467-023-38819-5)
Supplement: Supplementary file 1 — Supplementary Information [file 41467_2023_38819_MOESM1_ESM.pdf]

Supplementary Information

Photonic crystals with rainbow colors by centrifugation-assisted assembly of colloidal lignin nanoparticles

*Jinrong Liu,[1,2] Mathias Nero,[1] Kjell Jansson,[1] Tom Willhammar,[1] Mika H. Sipponen[1,2]**

[1]Department of Materials and Environmental Chemistry, Stockholm University, Sweden

[2] Wallenberg Wood Science Center, Department of Materials and Environmental Chemistry, Stockholm University, SE-10691 Stockholm, Sweden

This PDF contains:

Supplementary Table 1

Supplementary Figures 1−8

Supplementary References

Supplementary Tables

Supplementary Table 1. Calculation of the PDI of LNPs.

	Fig. 2e	Fig. 4d	Fig. S8d	Fig. S8c	Fig. S8b	Fig. S8a	Fig. 4c
**Peak (nm)**	/	240	200	180	170	160	150
**Average size (D) (nm) ***	77.4	226.8	193.0	190.7	175.5	156.0	154.2
**Standard deviation (σ) (nm)**	20.3	46.7	30.1	30.4	27.1	17.7	14.5
**PDI=$(\sigma/D)^2$**	0.069	0.042	0.024	0.020	0.030	0.013	0.009

* 100 particles were analysed.

Supplementary Figures

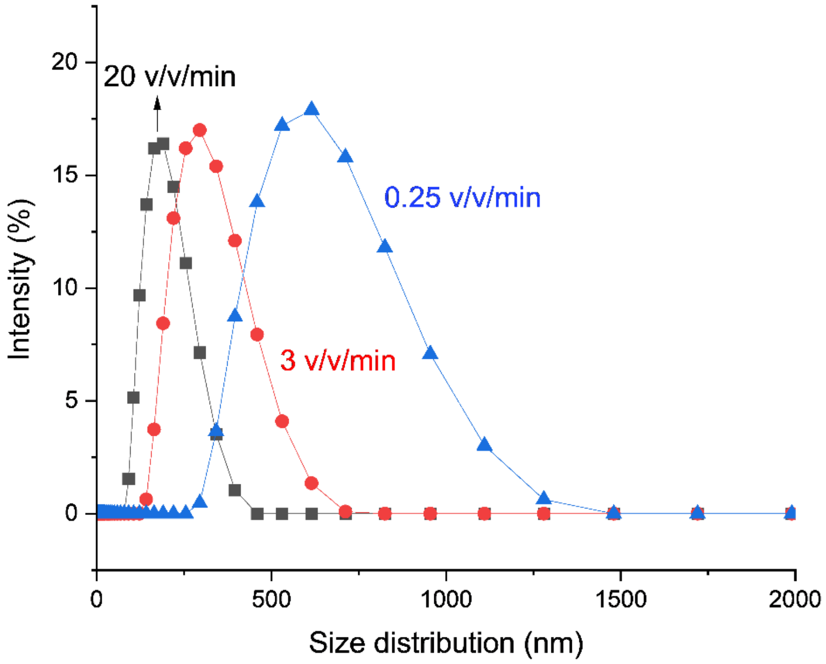

Supplementary Figure 1. DLS size distributions of LNP dispersion prepared at three different dilution rates by adding water to the lignin solution in a binary water-acetone solvent mixture.

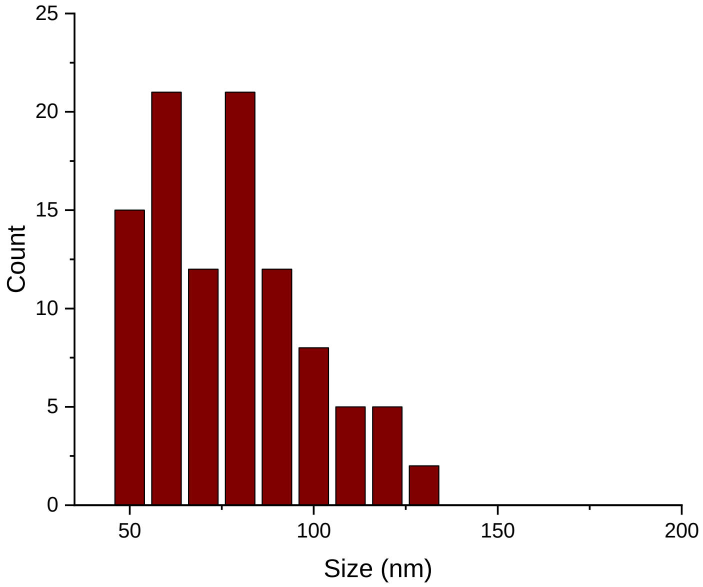

Supplementary Figure 2. Size distribution of LNPs measured from SEM image with PDI of 0.07. $PDI = \left(\frac{\sigma}{D}\right)^2$ [1], where the average size D is 77 nm, and the standard deviation σ is 20 nm.

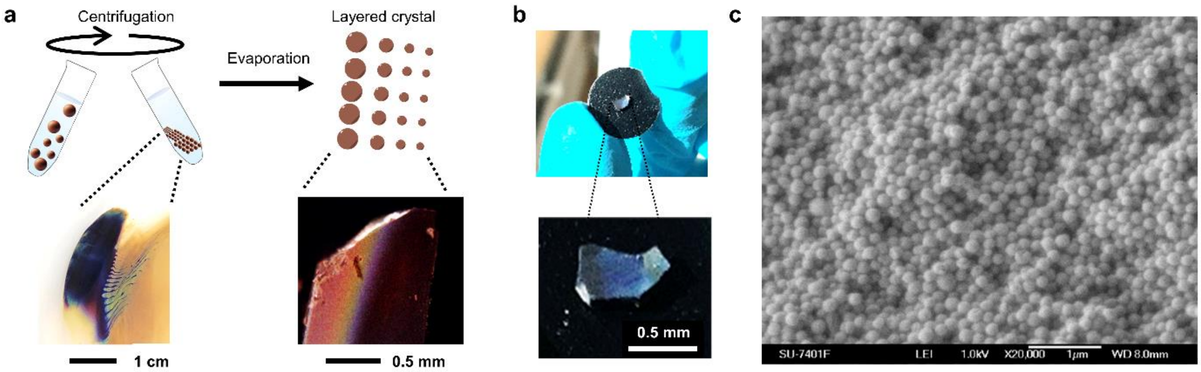

Supplementary Figure 3. (a) Scheme of forming photonic crystal from LNPs by centrifugation. Digital photo of the pellet after centrifugation (left) and digital microscope image of cross-section of LNPs photonic crystal (right). (b) Digital photograph of the photonic crystals. (c) SEM image of the cross-section of the photonic crystals.

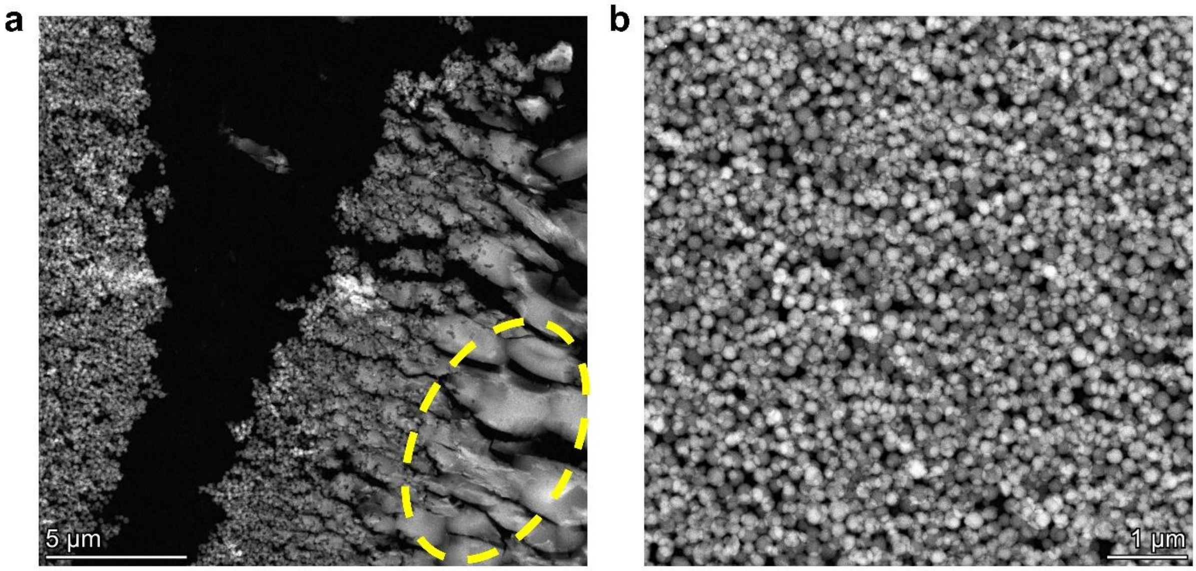

Supplementary Figure 4. (a) STEM image of sectioned LNPs photonic crystal. The region of resin was highlighted by a yellow circle. (b) STEM image of sectioned LNPs photonic crystal, which displays how the LNPs are arranged and packed inside the photonic crystal.

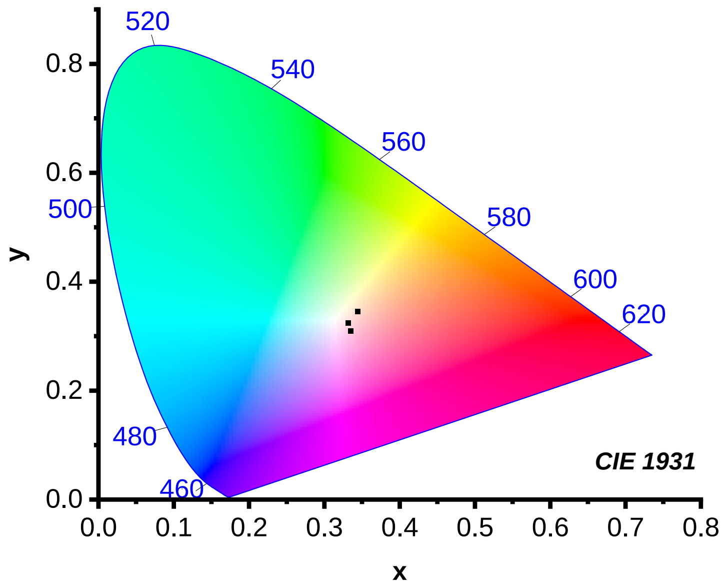

Supplementary Figure 5. CIE 1931 chromaticity coordinates of LNPs photonic crystal. Spectral power distribution of illuminant was D65.

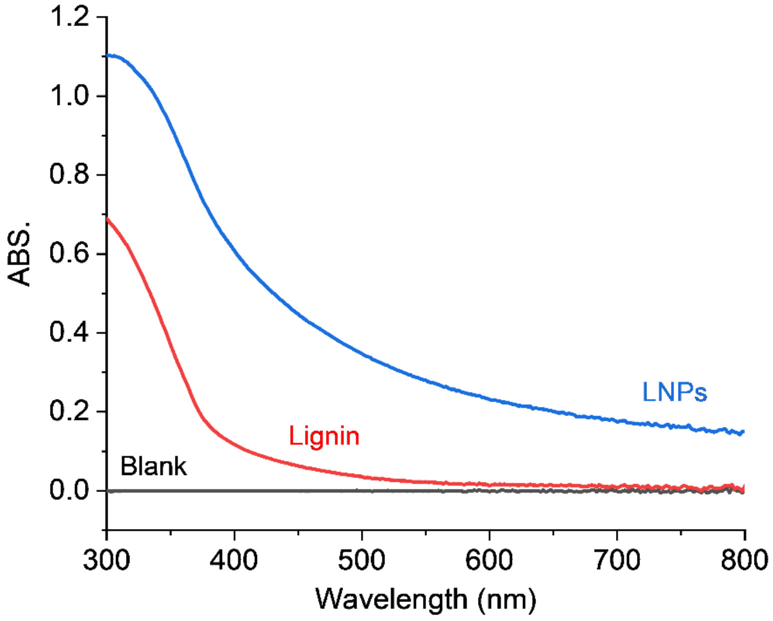

Supplementary Figure 6. Absorption spectra of lignin and LNPs. Lignin solution and LNPs dispersion were coated on a microscope cover glass. The spectrum of the cover glass was recorded as a background.

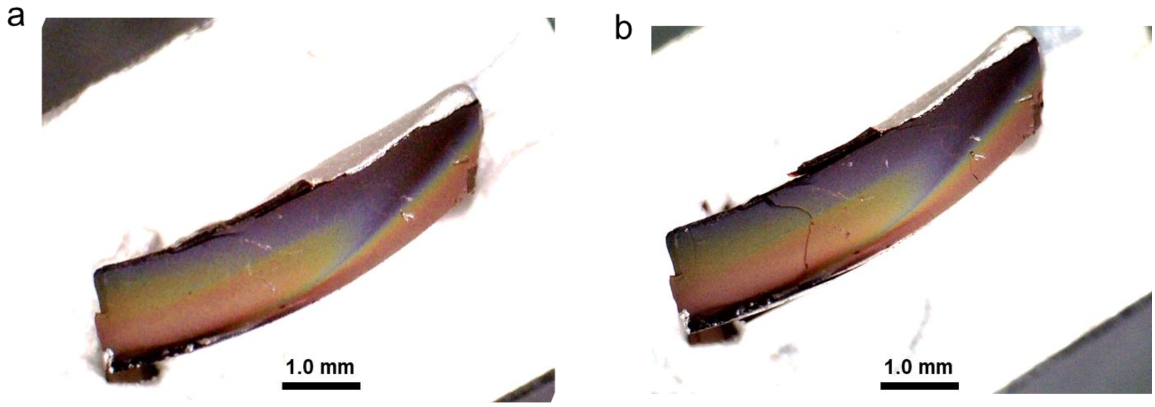

Supplementary Figure 7. Digital microscope images of LNP-PC (a) before and (b) after 120 s coating with gold.

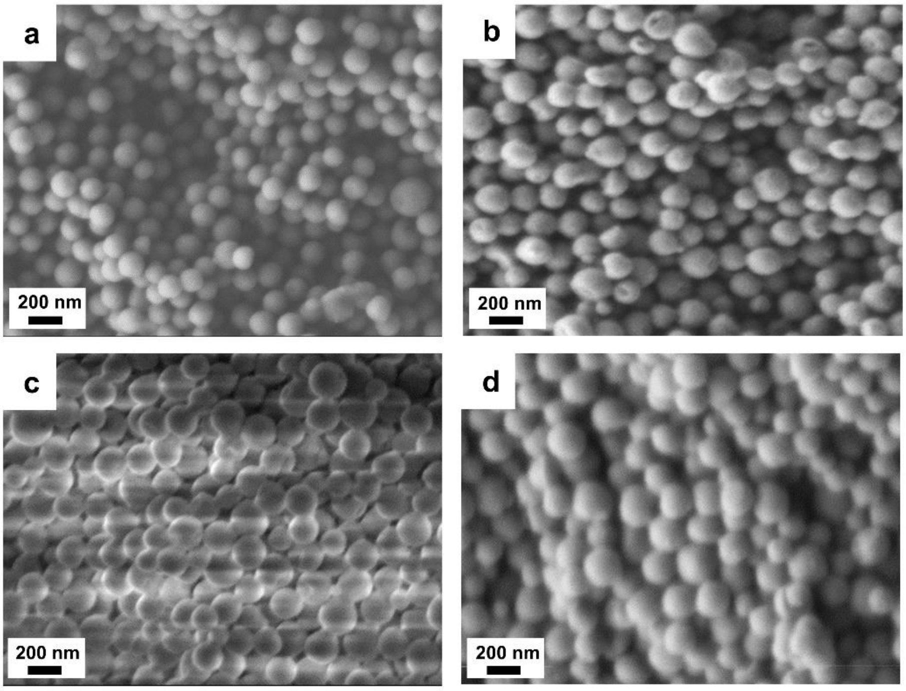

Supplementary Figure 8. SEM images of the fracture cross-section of LNPs in different color layers, (a) in blue, (b) in green, (c) in yellow, and (d) in orange.

Supplementary References

[1] N. Raval, R. Maheshwari, D. Kalyane, S.R. Youngren-Ortiz, M.B. Chougule, R.K. Tekade, Importance of physicochemical characterization of nanoparticles in pharmaceutical product development, Elsevier Inc., 2018.
