# Supplementary material for: Photonic crystals with rainbow colors by centrifugation-assisted assembly of colloidal lignin nanoparticles
Source: Nat Commun. 2023 May 29;14:3099. doi: 10.1038/s41467-023-38819-5 (PMC10227086; doi:10.1038/s41467-023-38819-5)
Supplement: Supplementary file 3 — Description of Additional Supplementary Files [file 41467_2023_38819_MOESM3_ESM.pdf]

Description of Additional Supplementary Files

File Name: Supplementary Movie 1
Description: Real-time recording of the evaporation-induced self-assembly of LNPs.
